# Improving the inclusion and participation of children and adolescents with a migration background in KiGGS Wave 2

**DOI:** 10.17886/RKI-GBE-2018-034

**Published:** 2018-03-15

**Authors:** Laura Frank, Rahsan Yesil-Jürgens, Sabine Born, Robert Hoffmann, Claudia Santos-Hövener, Thomas Lampert

**Affiliations:** 1 Robert Koch Institute, Berlin, Department of Epidemiology and Health Monitoring; 2 Charité – Universitätsmedizin Berlin, Department of Psychiatry and Psychotherapy

**Keywords:** MIGRATION, CHILDREN, ADOLESCENTS, HEALTH MONITORING, KIGGS

## Abstract

In the context of health monitoring at the Robert Koch Institute, the baseline study of the German Health Interview and Examination Survey for Children and Adolescents (KiGGS) surveyed a sample of children and adolescents with a migration background according to their share within the general population through extensive measures. Owing to less comprehensive efforts, this was not accomplished in the follow-up KiGGS Wave 1 study. For KiGGS Wave 2, the objective therefore was, through targeted measures, to increase the willingness of children and adolescents with a migration background to participate in the survey. This article describes the approaches to include children and adolescents with a migration background, the operationalisation of migration-specific variables and the effectiveness of field visits prior to the actual survey as a tool to increase the willingness of these groups to participate in the survey. Furthermore, data on participation and the sample of children and adolescents with a migration background in the cross-sectional KiGGS Wave 2 study is presented.

Overall, 2,994 children with a migration background aged 0 to 17 years took part in KiGGS Wave 2. In the weighted sample this corresponds to 11.8% (n=1,436) with a one-sided and 17.0% (n=1,558) with a two-sided migration background. In sum, the share of children and adolescents surveyed with a migration background (28.8%) is almost that of their share in Microcensus 2013 (31.2%). Compared to children and adolescents without a migration background, barely any differences exist in age and gender distribution, while differences are seen regarding social status; children with a two-sided migration background are significantly more often found in the low social status group. In the sample, the most often represented countries of origin were the countries of Central and South Europe, of the former Soviet Union and Turkey. Regarding the length of time parents had lived in Germany, around 40.1% of migrant families have been living in the country for over 20 years, whereas nearly one in five families has been in Germany for less than five years. A total of 12.2% of children and adolescents with a migration background migrated themselves.

By implementing a comprehensive set of measures, the degree after weighting to which children and adolescents with a migration background were included in KiGGS Wave 2 is nearly commensurate to their share in the overall population.

**KiGGS Wave 2**

Second follow-up to the German Health Interview and Examination Survey for Children and Adolescents

**Data owner:** Robert Koch Institute

**Aim:** Providing reliable information on health status, health-related behaviour, living conditions, protective and risk factors, and health care among children, adolescents and young adults living in Germany, with the possibility of trend and longitudinal analyses

**Study design**: Combined cross-sectional and cohort study

**Cross-sectional study in KiGGS Wave 2 Age range:** 0-17 years

**Population:** Children and adolescents with permanent residence in Germany

**Sampling:** Samples from official residency registries - randomly selected children and adolescents from the 167 cities and municipalities covered by the KiGGS baseline study

**Sample size:** 15,023 participants

**KiGGS cohort study in KiGGS Wave 2**

**Age range:** 10-31 years

**Sampling:** Re-invitation of everyone who took part in the KiGGS baseline study and who was willing to participate in a follow-up **Sample size:** 10,853 participants

**KiGGS survey waves**

► KiGGS baseline study (2003-2006), examination and interview survey
► KiGGS Wave 1 (2009-2012), interview survey
► KiGGS Wave 2 (2014-2017), examination and interview survey

More information is available at www.kiggs-studie.de/english

## 1. Introduction

In 2016, around 18.6 million people in Germany had a migration background which implies that they themselves or at least one of their parents have migrated to Germany [1]. This is 22.5% of the population. In the under 18 age group, one in three people has a migration background. People in Germany with a migration background are on average significantly younger than the population without a migration background. The share is therefore highest in the age group of children under five (38.1%) [1]. Migration visibly marks the living conditions and health even for second or third generation immigrant children in Germany. Based on the results of the baseline study of the German Health Interview and Examination Survey for Children and Adolescents (KiGGS), children and adolescents with a migration background show differences regarding physical and psychosocial health, health behaviour and health system utilisation compared to their peers without a migration background [2]. The health opportunities and disease risks vary depending on country of origin and length of stay, but also depend on age, gender and socioeconomic status [2].

To assess the health of people with a migration background requires a clear definition of migration background as a concept in the corresponding data [3]. Many official statistics, as well as routine data, however, only survey nationality as a differentiating factor. Nationality, however, can only show a part of the population with a migration background. Such an approach fails to identify the migration background of, for example, ethnic Germans from Eastern Europe (German resettlers) and naturalised foreigners, as they hold German citizenship. Health surveys at the Robert Koch Institute therefore apply the criterion of country of birth of participants and/or their parents. People who were born in Germany then can nonetheless be assigned a migration background, regardless of their current nationality [2, 4, 5].

Overall, there is still only insufficient data on the health of people with a migration background [6, 7]. People with a migration background are generally systematically under-represented in health surveys, because for different reasons their willingness to participate in such surveys is lower than that of the population without a migration background [8]. Language is one barrier to participation, as can be specific cultural factors, or fears that the health data surveyed could potentially be accessible to migration offices and therefore influence the decision on participants’ residence status [9, 10]. Therefore, special measures are required to include people with a migration background in health surveys. So far, only the baseline study of German Health Interview and Examination Survey for Children and Adolescents (KiGGS) provides national representative data [11-13] that allows a detailed description of health by migration background [2].

The KiGGS baseline study (examination and interview survey, 2003-2006) was the first survey to develop and implement an approach that specifically takes migration into account [14]. The share of participants with a migration background in the weighted sample was 25.9% (unweighted 22.1%) [15]. The first follow-up survey (KiGGS Wave 1, 2009-2012) was conducted as a telephone survey and the share of children and adolescents with a migration background was 24.3% (unweighted 16.3%) in the cross-sectional sample [15]. These low levels of participation are also due to the fact that interviews were conducted exclusively in German. Translated self-administered paper questionnaires were the only alternative offered and were used by 1.2% of parents in the cross-sectional sample. There were no home visits conducted as specific recruitment measure [16]. The second follow-up survey (KiGGS Wave 2, 2014-2017), which consisted of an interview and an examination, therefore strove to again take special measures to better represent children and adolescents with a migration background.

This article describes the measures taken to include children and adolescents with a migration background in the cross-sectional KiGGS Wave 2 study, the operationalisation of migration-specific variables and the efficiency of field visits prior to the survey as a measure to increase the willingness of people who are not German nationals to participate in the survey. Furthermore, data on levels of participation and makeup of the sample of children and adolescents with a migration background in the cross-sectional KiGGS Wave 2 study is presented.

## 2. Methodology

### 2.1 Study design

The KiGGS surveys regularly provide national data from Germany to describe the health of children and adolescents under 18 and can therefore reveal trends [12, 13, 16]. Since 2009, in the context of health monitoring at the Robert Koch Institute, KiGGS has been continued as a long-term survey. KiGGS surveys data on health status, health behaviour, living conditions, protection and risk factors as well as healthcare service utilisation. The survey concept, sampling and design as well as KiGGS Wave 2 implementation have been previously described in detail [17, 18]. Whereas all participants were interviewed, only some of the children and adolescents were subsequently also medically examined. This edition also contains a detailed description of response rates and sample composition [17].

All surveys at the Robert Koch-Institute are subject to strict compliance with the data protection regulations of Germany’s Federal Data Protection Act. Hannover Medical School´s ethics committee has considered and approved the survey under ethical guidelines (No. 2275-2014). The Federal Commissioner for Data Protection and Freedom of Information in Germany received the KiGGS Wave 2 study concept and had no objections. Together with the invitation to the survey, participants, their parents and/or legal guardians were informed on those responsible for the survey, the objectives and content of the survey, voluntary participation and data protection. They provided their informed consent in writing.

### 2.2 Measures to include children and adolescents with a migration background

**Figure 1:**
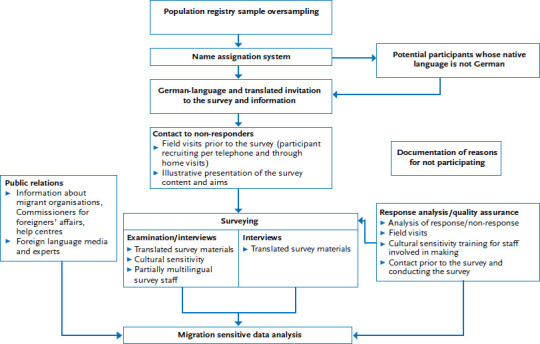
The KiGGS Wave 2 approach to account for migration as a factor Source: Based on Schenk et al. 2007 [14]

Based on the experience of previous KiGGS survey waves [2, 15], KiGGS Wave 2 continues and enhances the approach of the KiGGS baseline study [2] that specifically focused on including people with a migration background in a multi-step approach (Figure 1). To compensate for the low level of willingness of people with a migration background to participate in the survey, sampling involved the application of an oversampling factor of 1.5. The share of children and adolescents without German nationality in the unadjusted gross sample was therefore higher than their share in the population. Invitations to the survey and questionnaires were translated into four languages (Turkish, Russian, Serbo-Croatian and English). The selection of these languages was based on the size of the language group, the extent of the language difficulties observed, as well as the experiences from the KiGGS baseline study and KiGGS Wave 1 [2, 15]. A computer-based system to assign names developed by the Humpert und Schneiderheinze GbR (H&S) (onomastic procedure) was used that allows to assign first and last names of children and adolescents to specific languages and relate these to a possible migration background. Parents or legal guardians were then sent an invitation in German as well as in the language determined by the onomastic procedure [19, 20]. Due to ethical and legal regulations, the parents were the contact persons for concerns regarding the study [17]; following we use the term “family” when addressing survey-relevant connections between children and their parents. Families in the Arabic speaking group received an invitation in English, because no invitation and survey materials in Arabic were available.

A further measure for all families consisted in field visits prior to the survey [17]. Families that did not react to invitations and reminder letters were contacted first by telephone and, where necessary, contacted at their homes. In a personal conversation, survey staff provided these families with information on the objectives and content of the survey and answered any questions. Approaching families with a migration background in this manner aimed to increase their willingness to participate in the survey. In cases, where families could not be motivated to participate, survey staff attempted to determine their reasons for not participating. If contacting a person to clarify the objectives and contents of the survey proved impossible due to language barriers, these children and adolescents were counted as a quality neutral loss [17].

Examinations were culturally sensitive, and girls, for example, were examined only by female survey staff. To ensure participation by people with only a rudimentary knowledge of German, survey materials and consent forms were made available in four languages. Some field team staff were multilingual and this aimed to reduce language barriers in the examination centres.

Furthermore, response and non-response analyses were conducted that specifically took into account migration background and the response rate of non-German nationals was constantly monitored. Where necessary, efforts to increase these groups’ willingness to participate in the survey could be stepped up in a targeted manner. For example, field visits prior to the survey to contact people with a migration background were stepped up. Moreover, field staff and examination teams received cultural awareness training for the purpose of quality assurance. To systematically record the language or cultural difficulties faced by people with a migration background, a questionnaire was developed for staff involved in field visits prior to the survey and the teams that then conducted the actual survey.

Public relations activities were also conducted and, as potential multipliers to recruit participants, migrant organisations, commissioners for foreigners’ affairs and help centres at the 167 sample points were informed about the survey and its aims. All of these measures were important to ensure an analysis of data capable of accounting specifically for migration.

Due to the increase in immigration to Germany in particular in 2015, the delivered addresses from the population registry samples more often included families from crisis areas such as Syria or Iraq. With greater frequency, the survey staff involved in contacting potential participants prior to the survey documented that the people they had written to lived in centralized homes or in centres for unaccompanied minor refugees, which had made communication very difficult. Therefore, in the cross-sectional KiGGS Wave 2 study an additional survey-methodological module was developed to test accessing asylum seeker families. A short questionnaire was created and translated into Arabic and English. It was subsequently sent to all Syrian, Iraqi or Eritrean families that had not previously explicitly stated that they did not want to participate in the survey or who, due to language barriers, had been unable to participate in the survey (quality neutral losses).

### 2.3 Operationalisation of migration-specific variables

A participant’s migration background was established, as in previous KiGGS waves, based on the country of birth of a child or adolescent and where applicable that of their parents, as well as parent nationality [2]. A one-sided migration background was defined as having one parent not born in Germany or without German citizenship. The group of two-sided migration background included children who had themselves migrated to Germany and have at least one parent who was not born in Germany. Children and adolescents whose parents were both born in a country other than Germany or are non-German nationals also fall into this group, whether or not they themselves have migrated or were born in Germany. In single-parent households, the status of the single parent is the defining factor for child migration background.

All immigrants to Germany were asked what “immigration group” they belonged to. The following categories were provided: 1) asylum seekers, 2) recognised asylum seekers, 3) war refugees, 4) contingent refugees, 5) EU citizens, 6) family reunification, 7) labour migrants, 8) ethnic Germans from Eastern Europe, 9) students and 10) other.

The countries of origin were established based on parental country of birth and/or nationality. In families where the mother and father come from different countries, the mother’s country was taken. The fathers’ data was taken in cases where mothers failed to provide the corresponding data. The data on countries of origin was differentiated for further analyses if a sufficiently large number of people came from one particular country. Countries of origin for only small numbers of participants were regionally aggregated: 1) Germany, 2) Turkey, 3) countries of the former Soviet Union, 4) Poland, 5) Central and Southern Europe, 6) Canada, US, Israel and the rest of Europe, 7) Arabic countries and North Africa, 8) Latin America, 9) Asia and 10) sub-Saharan Africa (see Annex 1).

The year a participant’s mother had entered Germany defines the length of time parents have lived in Germany. If this date was unknown or the mother had been born in Germany, the father’s data was used for calculation. Participants were subdivided into five groups regarding length of stay: 0-5 years, 6-10 years, 11-15 years, 16-20 years and over 20 years.

All immigrants to Germany were asked whether they held permanent residency status. Parent residency status was initially established based on the data provided by mothers. Where mothers did not provide this information, were German or EU citizens, the data provided by the father was used. This system therefore differentiates between 1) Germans/EU citizens and 2) permanent and 3) temporary residency status.

Children and adolescents, who were not born in Germany, belong to the first generation. The second and subsequent generations comprise children and adolescents, who have lived in Germany since birth with at least one parent having been born outside Germany or without German citizenship.

### 2.4 Statistical analysis

Response rates and the efficiency of field visits prior to data collection were calculated based on the citizenship of children and adolescents delivered by the population registries. Migration background, which was established from the data on children, adolescents as well as their parents collected in the health questionnaires, then provided the basis for all further analyses.

In a first step, the share of children and adolescents with a migration background in KiGGS Wave 2 was verified based on the Microcensus 2013 distribution. As a mandatory representative household survey and part of official statistics in Germany, the Microcensus also includes data on people with a migration background [1, 21]. However, the Microcensus measures and defines migration background [22] differently to the KiGGS survey so the Microcensus 2013 data had to be adapted to fit the definition used in KiGGS. Corresponding shares for onesided and two-sided migration background were then calculated. A total of 2% of the people that the Microcensus defines as having a migration background do not fall into this category based on the definition applied by the KiGGS study.

A description of weighting in the KiGGS sample is provided in Hoffmann et al. [17]. To ensure representative assessments, KiGGS data was weighted regarding foreigner status (German citizenship yes/no) on the basis of population statistics (as at 31 December 2014) [23], as well by age, gender, parents’ levels of education and federal state. Hoffmann et al. describe the weighting procedure in KiGGS Wave 2. The descriptive analyses of the cross-sectional sample of children and adolescents with a migration background stratified by age, socioeconomic status and size of home town were conducted with and without weighted KiGGS data. To assess the influence of weighting on migration background and other migration-specific variables, case numbers and frequency are presented unweighted and weighted.

## 3. Results

### 3.1 Participation of children and adolescents with a migration background

The response rate of children and adolescents with non-German nationality was 17.0% overall, within the examination sample it was 27.9%. In the unweighted KiGGS sample, the share of children and adolescents of non-German nationality was 3.7%. Weighting increased this share to 7%, which is in line with the population figures from the Federal Statistical Office (7.0%) [23].

Out of a total of 15,023 participants in the cross-sectional KiGGS Wave 2 study, 2,994 children and adolescents have a migration background (Table 1). Overall, 1,436 children had a one-sided migration background, which, in the weighted sample, corresponds to a share of 11.8% (unweighted 9.7%). A total of 17.0% (weighted) of children had a two-sided migration background (unweighted 10.5%). In Microcensus 2013 data, 10.7% have a one-sided and 20.5% a two-sided migration background. Overall, the weighted share of children and adolescents with a migration background (28.8%) in KiGGS Wave 2 is therefore nearly commensurate to their Microcensus 2013 share (31.2%).

Additionally, as part of the survey-methodological module on accessing asylum-seeking families subsequent to the KiGGS study, the short questionnaire was handed out to 402 Syrian, Iraqi and Eritrean nationals living in Germany. The questionnaire could not be delivered to 65 of them. The response rate was therefore 19.0% (n=64).

### 3.2 Field visits prior to the survey to contact non-German nationality families

**Figure 2:**
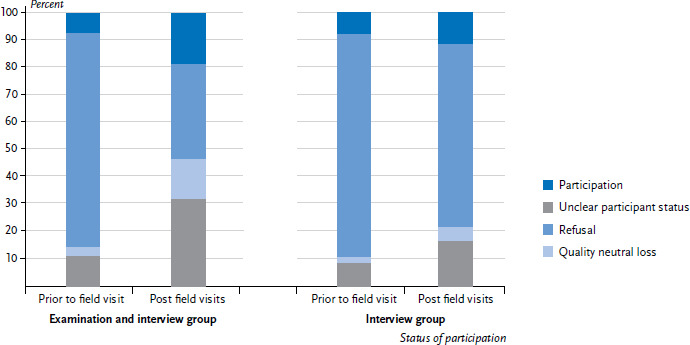
Increase of participation among families of non-German nationality due to visits prior to the survey (interview and examination n=482 girls, n=543 boys; interview n=1,624 girls, n=1,841 boys) Source: KiGGS Wave 2 (2014-2017)

An important measure to include people with a migration background in the survey consisted of contacting them prior to the survey. No analysis of the effectiveness of the other measures described under section 2.3 is provided because the effects of these measures cannot be clearly defined separately. Figure 2 shows the shares of invited sample members’ willingness to participate both before the field visits and after the data collection had finished. However, the shown results cannot be clearly assigned to the field visits, because changes in the willingness to participate could also have appeared (after the field visits and prior to the actual participation) independently of the field visits. Results are based on data of the gross sample of people without German citizenship. Among the examination sample (n=1,025), the share of cases with unknown participant status was reduced from 77.8% after the postal reminder to 34.7% after the end of data collection. This implies that the share of participating sample members doubled (from 7.9% to 19.0%) after field visits. Both the shares of refusals and quality neutral losses tripled. Among the interview sample (n=3,465), field visits were generally conducted less extensively than in the examination group [17].This also applies for the sample members without German citizenship (interview sample n=3,465). The share of cases with unclear participant status was 66.8% and therefore approximately twice as high as in the examination group. The share of participants in the gross sample increased from 8.3% to 11.8%. Both the proportion of refusals and quality neutral losses doubled. On the whole, there were less observable status changes in the interview sample than in the examination sample.

### 3.3 Socio-demographic and socioeconomic differences between children and adolescents regarding migration background

Regarding age and gender distribution hardly any differences to children and adolescents without migration background exist compared to children and adolescents with a migration background. In the unweighted sample, regardless of migration background, the share of small children aged 0 to 2 years is the lowest compared to other age groups. There were no examinations conducted in this age group. Weighting partially offset this difference. The share of children and adolescents with a two-sided migration background was highest in the 14 to 17 age group (Table 2).

Clear social differences are however apparent (Table 2). Children and adolescents with a two-sided migration background more often fall into the lower social status group (45.1% versus 13.6% without a migration background) and less in the high status group (7.7% versus 22.6% without a migration background). The social composition of the group of children and adolescents with one-sided migration background thereby tends to resemble that of their peers without a migration background. Equally, a clear urban-rural differential is evident, with a greater share of children and adolescents with a one-sided or two-sided migration background living in larger cities than in rural areas (Table 2).

### 3.4 Differentiating children and adolescents within the larger group with a migration background

Differentiated on the basis of their mothers and fathers, ethnic Germans from Eastern Europe, people with a migration background, who came to Germany through family reunification regulations and EU citizens, comprise the largest immigrant groups represented in KiGGS Wave 2 (Table 3). Compared to the KiGGS baseline study, the share of asylum seekers and war refugees has increased in KiGGS Wave 2 [2].

Due to the information potentially available about particular cultural backgrounds or a country of origin’s health system, parental country of birth is an important stratification variable. Most frequently the children and adolescents with a migration background had family ties to Central and Southern Europe (6.0%), the countries of the former Soviet Union (5.4%) and Turkey (4.2%) (Table 4). Considering the length of stay of parents, around 40% of migrant families have been living in Germany for over 20 years. By contrast, one in five migrant families has been in Germany for less than five years (Table 4). A total of 12.2% of children and adolescents with a migration background have migrated themselves. The majority of children and adolescents has a secure residency status, yet 11.9% of families have only temporary residency status and correspondingly only an uncertain perspective of whether they will be able to remain in Germany (Table 4). Participants spoke 72 languages at home in sum. Among these, the languages most frequently mentioned were Russian (16.9%), Turkish (16.6%), Polish (8.0%), Arabic (6.8%) and English (5.7%).

## 4. Discussion

The results show the success of intensified measures to motivate families with a migration background to participate in KiGGS Wave 2 and the weighted share of children and adolescents with a migration background is almost commensurate to this group’s share in the overall population. However, whereas sufficient children and adolescents with a one-sided migration background participated in the cross-sectional KiGGS Wave 2 study, the unweighted KiGGS sample underrepresented children and adolescents with a two-sided migration background. Weighting approximately compensated for these differences in distribution with regard to Microcensus 2013 data. However, even after weighting, distortions concerning other variables such as length of stay or country of origin may subsist because weighting does not consider these variables. A clear limitation of the analysis is the diverging definitions of migration background, which means that full comparability with Microcensus data is not possible. This, however, only affected a very small number of people, who could not be unambiguously categorised according to the Robert Koch Institute definition. Moreover, the survey excluded people who did not speak sufficient German or who did not speak one of the four languages into which the survey materials and consent forms had been translated and which clarified the survey’s aims and contents (informed consent). In particular, participation by presumably Arabic speaking families that appeared in the gross sample was limited.

Contacting and including possibly asylum-seeking families was tested using an abbreviated questionnaire that went to Syrian, Iraqi and Eritrean families following the standardised survey procedures. This additional survey-methodological module provided information on possible barriers to including asylum seekers in the survey. Nearly one in five families could not be contacted at their address. Over and above the language barrier, the greatest difficulty in contacting and including this group in KiGGS Wave 2, therefore, was the group’s mobility (for example due to their re-distribution to other centres or places of accommodation). A limiting factor in this analysis is that not all families with Syrian, Iraqi or Eritrean citizenship are asylum-seekers.

Because various measures were being applied simultaneously, the efficiency of individual migration-specific measures cannot be clearly established. Specially trained survey staff, however, who had established a personal contact with families prior to the survey, proved an effective way to increase willingness to participate. In particular for the examination sample, willingness to participate in the survey doubled. Furthermore, the response rate was nearly twice as high as in the interview sample, where field visits prior to the survey were not conducted as intensively. As other surveys have revealed, while time-consuming, personal contact is a necessary effort to convince people with a migration background to participate in surveys [3, 8, 24-26].

As in the KiGGS baseline study, the response rate of non-German nationals in KiGGS Wave 2 [2] was lower than for children and adolescents without a migration background [17]. This highlights the importance of implementing a broad set of measures to ensure participation by people with a migration background to adequately represent sub-populations that are particularly hard to reach, such as people who have limited German language skills.

To date, the KiGGS study in Germany remains the only cross-sectional health survey to include a sufficiently large number of people with a migration background. In particular, the possibilities to compare health-related markers of children and adolescents with and without a migration background promise valuable scientific findings [2]. The survey data could help close some of the current information gaps on the health of children and adolescents with a migration background and to conduct an analysis of trends. Next to cross-sectional analysis, we will analyse and present the potential of analyses of trends and possibly longitudinal analyses regarding people with a migration background within the context of KiGGS [27]. Regarding the diversity of German society, the measures described to increase the participation of people with a migration background in health statistics are truly indispensable.

The Robert Koch Institute, based on the ‘Improving Health Monitoring in Migrant Populations’ (IMIRA) project, therefore is currently conducting a diverse set of measures to improve the data (which is in many cases still only fragmentary) and information on people with a migration background [28]. One priority is the expansion of health monitoring at the Robert Koch Institute. In order to include adults with a migration background in Robert Koch Institute surveys in the long term, two feasibility studies will be conducted. Within the context of the feasibility study “interview”, new forms of approaching and measures to recruit participants will be tested, the content and surveying instruments reviewed and, where necessary, updated. The feasibility study “examination”, moreover, will test different options to reduce language barriers and difficulties between participants and the medical staff conducting examinations. Also, the project aims to expand health reporting regarding people with a migration background. In addition to health monitoring data, the aim is to increasingly use further sources of data such as that available from social insurers or the public health services.

In conclusion, it requires resource-intensive efforts to recruit a representative sample of the population with a migration background and collect data from a sufficiently large number of cases to answer migration-specific questions.

## Key statements

- After weighting children and adolescents with a migration background are represented in KiGGS Wave 2 nearly according to their share in the overall population.
- Personal contact through field visits by specifically trained survey staff is an effective measure of increasing willingness to participate in the survey.
- The countries of Central and Southern Europe, the former Soviet Union and Turkey were the most common countries of origin in the survey sample.
- Nearly one in five families with a migration background has been living in Germany for less than five years.
- A migration sensitive approach provides the basis for a representative sample and a migrant-specific data analysis.

## Figures and Tables

**Table 1:** Share of children and adolescents with a migration background in the cross-sectional KiGGS Wave 2 study compared to Microcensus 2013 Source: KiGGS Wave 2 (2014-2017), Microcensus 2013 [22]

	Cases, unweighted	Sample unweighted %	Sample weighted %	Microcensus 2013 in %
Without migration background	11,857	79.8	71.2	68.7
Total migration background	2,994	20.2	28.8	31.2
Among these:				
One-sided migration background	1,436	9.7	11.8	10.7
Two-sided migration background	1,558	10.5	17.0	20.5
Missing values	172	1.3		2.0

**Table 2:** Share of children and adolescents with a migration background by age, socioeconomic status and size of town in the cross-sectional KiGGS Wave 2 study (n=7,456 girls, n=7,395 boys) Source: KiGGS Wave 2 (2014-2017)

Migration background	Sample unweighted %			Sample weighted %		
	Without	One-sided	Two-sided	Without	One-sided	Two-sided
**Age (years)**						
0-2	9.3	12.8	10.4	14.9	21.8	14.3
3-6	22.6	26.0	21.2	21.0	24.5	21.1
7-10	23.3	23.9	22.7	21.5	21.1	21.6
11-13	20.6	18.4	19.3	17.7	15.1	14.8
14-17	24.3	18.9	26.4	24.9	17.6	28.2
Total	100	100	100	100	100	100
Missing values (n=0)						
**Socioeconomic status**						
Low	9.9	14.0	30.2	13.6	22.9	45.1
Medium	63.0	54.6	56.8	63.8	55.2	47.2
High	27.1	31.4	13.0	22.6	21.8	7.7
Total	100	100	100	100	100	100
Missing values (n=216)						
**Size of town**						
Rural	20.0	12.9	5.1	18.7	12.3	5.1
Small town	34.2	25.9	23.4	29.9	22.4	18.7
Medium-sized town	27.6	29.9	34.4	28.1	27.9	33.8
Large city	18.2	31.3	37.1	23.3	37.4	42.5
Total	100	100	100	100	100	100
Missing values (n=0)						

**Table 3:** Mothers and fathers of children and adolescents with a migration background by immigrant type in the cross-sectional KiGGS Wave 2 study Source: KiGGS Wave 2 (2014-2017)

	Mother		Father	
	Sample unweighted %	Sample weighted %	Sample unweighted %	Sample weighted %
Ethnic Germans	29.1	26.1	26.7	24.0
Family reunification	24.2	24.4	17.3	16.4
EU citizens	17.4	16.2	15.7	15.7
Asylum seekers	6.7	9.6	9.9	12.9
Other groups	8.1	8.2	8.6	8.4
War refugees	4.3	5.5	6.2	7.8
Labour migrants	4.0	4.7	7.1	7.6
Recognised asylum seekers	2.3	2.2	3.3	2.4
Students	2.9	2.1	4.2	3.6
Contingent refugees	1.0	1.1	1.1	1.1
Total	100	100	100	100

Missing values (Mother n=586, Father n=979)

**Table 4:** Migration-specific features of children and adolescents with a migration background in the cross-sectional KiGGS Wave 2 study (n=1,567 girls, n=1,433 boys) Source: KiGGS Wave 2 (2014-2017)

	Cases unweighted %	Sample unweighted %	Sample weighted %
**Country of origin**			
Germany	11,857	80.3	71.6
Turkey	332	2.2	4.2
Former countries of the Soviet Union	613	4.1	5.4
Poland	314	2.1	2.8
Central and Southern Europe	576	3.9	6.0
Canada, USA, Israel and the rest of Europe	450	3.0	3.3
Arab countries and North Africa	312	2.1	3.6
Latin America	64	0.4	0.5
Asia	164	1.1	1.5
Sub-Saharan Africa	93	0.6	1.0
Missing values	248		
**Length of stay in years**			
0-5	307	12.6	14.9
6-10	255	10.5	11.2
11-15	388	16.0	15.0
16-20	440	18.1	18.7
>20	1,042	42.8	40.1
Missing values	562		
**Residency status**			
Permanent	744	25.2	26.9
Temporary	267	9.0	11.9
German/EU citizen	1,944	65.8	61.2
Missing values	39		
**Immigrant generation**			
First generation	307	10.3	12.2
Second and subsequent generations	2,687	89.7	87.8
Missing values	-		

## Appendix

**Annex 1:** Countries of origin of children and adolescents with a migration background, KiGGS Wave 2 cross-sectional study Source: KiGGS Wave 2 (2014-2017)

Countries of origin:	
1)	Germany
2)	Turkey
3)	Countries of the former Soviet Union: the Soviet Union, Russia, Estonia, Latvia, Lithuania, Ukraine, Belarus, Uzbekistan, Kazakhstan, Georgia, Azerbaijan, Kyrgyzstan, Tajikistan, Turkmenistan, Armenia and Moldova
4)	Poland
5)	Arab countries and North Africa: Lebanon, Morocco, Algeria, Iraq, Egypt, Pakistan, Syria, Jordan, Tunisia, Iran, Kuwait and Sudan
6)	Southern Europe/Mediterranean: Albania, Bosnia, Bulgaria, Croatia, Slovenia, Greece, Italy, Yugoslavia, Macedonia, Spain, Portugal, Cyprus, Serbia, Kosovo, Romania and Montenegro
7)	USA, Australia, Canada, Israel and the rest of Europe: Belgium, Denmark, Finland, France, Ireland, Luxembourg, the Netherlands, Norway, Austria, Slovakia, Sweden, Switzerland, the Czech Republic, Czechoslovakia, Hungary, the United Kingdom and Iceland
8)	Latin America: Argentina, Brazil, Chile, Costa Rica, Dominican Republic, Ecuador, El Salvador, Colombia, Mexico, Nicaragua, Paraguay, Peru, Uruguay, Venezuela, Jamaica and Haiti
9)	Asia: Bhutan, Sri Lanka, Vietnam, India, Japan, Laos, Mongolia, Nepal, Philippines, Taiwan, Korea, Thailand, China, Malaysia, Cambodia, Brunei, Indonesia and Bangladesh
10)	Sub-Saharan Africa: Angola, Eritrea, Ethiopia, Nigeria, Ghana, Kenia, Congo, Liberia, Madagascar, Mauritius, Mozambique, Cameroon, South Africa, Namibia, Sierra Leone, Togo, Burkina Faso, Ivory Coast, Benin, Uganda, Cape Verde, Somalia, Senegal, Guinea and Gambia

## References

[1] Statistisches Bundesamt (2017) Bevölkerung und Erwerbstätigkeit. Bevölkerung mit Migrationshintergrund. Ergebnisse des Mikrozensus 2016. Fachserie 1 Reihe 2.2. Destatis, Wiesbaden

[2] Robert Koch Institute (Ed) (2008) Kinder- und Jugendgesundheitssurvey (KiGGS) 2003-2006: Kinder und Jugendliche mit Migrationshintergrund in Deutschland. Beiträge zur Gesundheitsberichterstattung des Bundes. RKI, Berlin. http://edoc.rki.de/documents/rki_fv/reJBwqKp45PiI/PDF/23Ydv-84JGTBo6_07.pdf (As at 23.02.2017)

[3] Reiss K Makarova N Spallek J (2013) Identifizierung und Rekrutierung von Menschen mit Migrationshintergrund für epidemiologische Studien in Deutschland. Gesundheitswesen 75(6):e49-58 22932826 10.1055/s-0032-1321768

[4] Robert Koch Institute (2008) Migration und Gesundheit. Schwerpunktbericht der Gesundheitsberichterstattung des Bundes. RKI, Berlin. http://edoc.rki.de/documents/rki_fv/ren4T3cctjHcA/PDF/253b-KE5YVJxo_28.pdf (As at 23.02.2017)

[5] Rommel A Saß AC Born S (2015) Die gesundheitliche Lage von Menschen mit Migrationshintergrund und die Bedeutung des sozioökonomischen Status. Erste Ergebnisse der Studie zur Gesundheit Erwachsener in Deutschland (DEGS 1). Bundesgesundheitsbl Gesundheitsforsch Gesundheitsschutz 58(6):543-552. http://edoc.rki.de/oa/articles/resmIGjZuxoiA/PDF/24KgGwHkF-gVzo.pdf (As at 23.02.2017) 10.1007/s00103-015-2145-2 25824135

[6] Robert Koch Institute (Ed) (2015) Gesundheit in Deutschland. Gesundheitsberichterstattung des Bundes Gemeinsam getragen von RKI und Destatis RKI, Berlin. http://edoc.rki.de/documents/rki_fv/refNzCggQ8fNw/PDF/29PIbXnI56Jfc.pdf (As at 23.02.2017)

[7] Frank L Rommel A Lampert T (2017) Die gesundheitliche Situation von Menschen mit Migrationshintergrund in Deutschland. GGW Jg. 17, Heft 2 (April), 7–14

[8] Ibrahim S Sidani S (2014) Strategies to recruit minority persons: a systematic review. J Immigr Minor Health 16(5):882-888 23338906 10.1007/s10903-013-9783-y

[9] Schenk L (2002) Migrantenspezifische Teilnahmebarrieren und Zugangsmoglichkeiten im Kinder- und Jugendgesundheitssurvey. Gesundheitswesen 64 Suppl 1:S59-68 12870218 10.1055/s-2002-39007

[10] Schenk L Neuhauser H (2005) Beteiligung von Migranten im telefonischen Gesundheitssurvey: Möglichkeiten und Grenzen. Gesundheitswesen 67(10):719-725 16235140 10.1055/s-2005-858655

[11] Hölling H Kamtsiuris P Lange M (2007) Der Kinder- und Jugendgesundheitssurvey (KiGGS): Studienmanagement und Durchführung der Feldarbeit. Bundesgesundheitsbl Gesundheitsforsch Gesundheitsschutz 50(5-6):557-566. http://edoc.rki.de/oa/articles/rej53eEjT1Ze6/PDF/29ruDT0W37I-rU.pdf (As at 23.02.2017) 10.1007/s00103-007-0216-8 17514439

[12] Kamtsiuris P Lange M Schaffrath Rosario A (2007) Der Kinderund Jugendgesundheitssurvey (KiGGS): Stichprobendesign, Response und Nonresponse-Analyse. Bundesgesundheitsbl Gesundheitsforsch Gesundheitsschutz 50(5-6):547-556. http://edoc.rki.de/oa/articles/rej53eEjT1Ze6/PDF/211Cul3e7Mh-kk.pdf (As at 23.02.2017) 10.1007/s00103-007-0215-9 17514438

[13] Kurth BM Kamtsiuris P Hölling H (2008) The challenge of comprehensively mapping children’s health in a nation-wide health survey: design of the German KiGGS-Study. BMC Public Health 8:196 18533019 10.1186/1471-2458-8-196 PMC2442072

[14] Schenk L Ellert U Neuhauser H (2007) Kinder und Jugendliche mit Migrationshintergrund in Deutschland. Methodische Aspekte im Kinder- und Jugendgesundheitssurvey (KiGGS). Bundesgesundheitsbl Gesundheitsforsch Gesundheitsschutz 50 (5-6):590-599. http://edoc.rki.de/oa/articles/reunJYxaLNDfs/PDF/233ll5mf-g7L5c.pdf (As at 23.02.2017) 10.1007/s00103-007-0220-z 17514443

[15] Sass AC Grüne B Brettschneider AK (2015) Beteiligung von Menschen mit Migrationshintergrund an Gesundheitssurveys des Robert Koch-Instituts. Bundesgesundheitsbl Gesundheitsforsch Gesundheitsschutz 58(6):533-542 10.1007/s00103-015-2146-1 25896496

[16] Lange M Butschalowsky HG Jentsch F (2014) Die erste KiGGS-Folgebefragung (KiGGS Welle 1): Studiendurchführung, Stichprobendesign und Response. Bundesgesundheitsbl Gesundheitsforsch Gesundheitsschutz 57(7):747-761. http://edoc.rki.de/oa/articles/re5weWnRsXRSw/PDF/20B6fVTP-FIdw.pdf (As at 23.02.2017) 10.1007/s00103-014-1973-9 24950824

[17] Hoffmann R Lange M Butschalowsky H (2018) KiGGS Wave 2 cross-sectional study – participant acquisition, response rates and representativeness. Journal of Health Monitoring 3(1):78-91. www.rki.de/journalhealthmonitoring-en (As at 15.03.2018) 10.17886/RKI-GBE-2018-032 PMC8848911 35586176

[18] Mauz E Gößwald A Kamtsiuris P (2017) New data for action. Data collection for KiGGS Wave 2 has been completed. Journal of Health Monitoring 2(S3):2-27. http://edoc.rki.de/oa/articles/revpaHQ3DqMU/PDF/25Pxmf2f-cHqRM.pdf (As at 27.09.2017) 10.17886/RKI-GBE-2017-105 PMC10291840 37377941

[19] Humpert A Schneiderheinze K (2000) Stichprobenziehung für telefonische Zuwandererumfragen. Einsatzmöglichkeiten der Namensforschung. ZUMA-Nachrichten 47:36-64

[20] Humpert A Schneiderheinze K (2002) Stichprobenziehung für telefonische Zuwandererumfragen. Praktische Erfahrungen und Erweiterung der Auswahlgrundlage. In: Gäbler S Häder S (Eds) Telefonstichproben Methodische Innovationen und Anwendungen in Deutschland Waxmann, Münster, P. 187-208

[21] Statistisches Bundesamt (2013) Bevölkerung nach Migrationshintergrund, Body-Mass-Index, Rauchverhalten, gesundheitlich bedingten Erwerbsunterbrechungen und Armutsgefähr-dungsquote. Daten aus dem Mikrozensus 2005 und 2009 (Sonderauswertung). Statistisches Bundesamt, Wiesbaden

[22] Research Data Centres of the Federal Statistical Office and Statistical Offices of the Länder (2016). Schlüsselverzeichnis Mikrozensus 2013. http://www.forschungsdatenzentrum.de/bestand/mikrozensus/gwap_kdfv/2013/fdz_mz_2013_schluesselverzeichnis.pdf (As at 27.09.2017)

[23] Research Data Centres of the Federal Statistical Office and Statistical Offices of the Länder (2017) Microcensus. 2013, own calculations. http://www.forschungsdatenzentrum.de/bestand/mikrozensus/(As at 20.11.2017)

[24] Aichberger M Yesil R Rapp MA (2012) Surveying migrant populations - methodological considerations: An example from Germany. Int J Cult Ment Health 1-15

[25] Chasan-Taber L Fortner RT Hastings V (2009) Strategies for recruiting Hispanic women into a prospective cohort study of modifiable risk factors for gestational diabetes mellitus. BMC Pregnancy and Childbirth 9:57-57 20003350 10.1186/1471-2393-9-57 PMC2799379

[26] Reiss K Dragano N Ellert U (2014) Comparing sampling strategies to recruit migrants for an epidemiological study. Results from a German feasibility study. Eur J Public Health 24(5):721-726 24872519 10.1093/eurpub/cku046

[27] Lange M Hoffmann R Mauz E (2018) KiGGS Wave 2 longitudinal component – data collection design and developments in the numbers of participants in the KiGGS cohort. Journal of Health Monitoring 3(1):92-107. www.rki.de/journalhealthmonitoring-en (As at 15.03.2018) 10.17886/RKI-GBE-2018-035 PMC8848915 35586182

[28] Robert Koch Institute, IMIRA-Forschungsprojekt. http://www.rki.de/DE/Content/Gesundheitsmonitoring/Themen/Migration/IMIRA/IMIRA_tab.html (As at 23.02.2017)

